# Supplementary material for: Non-host class II ribonucleotide reductase in Thermus viruses: sequence adaptation and host interaction
Source: PeerJ. 2019 Apr 8;7:e6700. doi: 10.7717/peerj.6700 (PMC6459318; doi:10.7717/peerj.6700)
Supplement: Supplemental Information 1 [file peerj-07-6700-s001.docx]

**Non-host class II ribonucleotide reductase in *Thermus* viruses: sequence adaptation and host interaction**

Loderer Christoph^1^*, Karin Holmfeldt^2^ Lundin Daniel^3^

^1^Institute for Microbiology, Technische Universität Dresden, Dresden, Saxony, Germany

ORCID: https://orcid.org/0000-0002-3795-8250

^2^Centre for Ecology and Evolution in Microbial model Systems - EEMiS, Linnaeus University, Kalmar, Sweden

ORCID: https://orcid.org/0000-0002-6887-6661

^3^Department of Biochemistry and Biophysics, Stockholm University, Stockholm, Sweden

ORCID: https://orcid.org/0000-0002-8779-6464e

Corresponding author: Christoph Loderer

Christoph.Loderer@tu-dresden.de

1. **Codon usage similarity**

The similarity of codon usage between Firmicutes, Thermus spp. and the viral nrdJm genes was calculated by euclidean distances between codon frequencies. The tables below show example values for this distances. Table S1 shows the similarities between the two viral *nrdJm* genes and different Thermus species. Table S2 shows the similarities between the different Firmicutes species and the *nrdJm* gene from TV P74-26 as well as between the Firmicutes and *Thermus thermophilus*.

|  | ***Thermus sp.*** *- TV P74-26* | ***Thermus sp.*** *- TV P23-45* |
| --- | --- | --- |
| *T. thermophilus* | 0.96 | 0.93 |
| *T. brockianus* | 0.80 | 0.75 |
| *T. oshimai* | 1.25 | 1.23 |

Table S1: Codon usage similarity as euclidean distances between different Thermus species and viral nrdJm genes.

|  | **Firmicute - TV P74-26** | **Firmicute - T. thermophilus** |
| --- | --- | --- |
| *C. perfringens* | 1.95 | 2.44 |
| *H. hemicellulosilytica* | 1.38 | 1.92 |
| *C. difficile* | 1.89 | 2.40 |
| *A. odorimutans* | 1.87 | 2.37 |
| *B. borstelensis* | 1.05 | 1.46 |
| *B. cereus* | 1.66 | 2.17 |

Table S2: Codon usage similarity as euclidean distances between different Firmicutes species and the TV P74.26 nrdJm gene and Thermus thermophilus, respectively.

1. **Phylogenetic tree accession numbers**

Tables S3-S6: Accession numbers for sequences in the phylogeny in Figure 1A and B. IMG metagenome, SAG and MAG accessions used for the search for metagenomic sequences fitting the TV/Firmicute NrdJm clan.

Supplemental files: Table_S3_Figure_1A_Phylogeny_Accessions.xlsx

Table_S4_IMG_MAG_Accessions.xlsx

Table_S5_IMG_Metagenome_Accessions.xlsx

Table_S6_IMG_SAG_Accessions.xlsx

1. **Temperature and GC content**

Table S7: Organism characteristics used for the heat maps in Figure 1B.

Supplemental file: Table_S7_Figure_1B_GC_Temperature.xlsx

1. **Best alignment of the TV P74-26 proteome**

| **Phylum** | **Number of best matches** |
| --- | --- |
| Deinococcus-Thermus | 8 |
| Firmicutes | 6 |
| Proteobacteria | 11 |
| Actinobacteria | 3 |
| Other | 6 |

Table S8. Similarity of viral sequences. Phylum of best alignment of the full TV P74-26 proteome when compared to the NCBI RefSeq database.

To get an overview over the origin of the gene from Thermus virus P74-26, we made a blast search with the full proteome of the virus and collected the best hits. The original table, which is the basis for the count in Table S8 is given in Table S9.

Table S9: Best alignment of Thermus phage P74-26 proteins to the NCBI RefSeq database with annotated function, identity of the best match and data of the alignment.

Supplemental file: Table_S9_Proteome_Best_Match.xlsx

1. **Investigation of non-conservative mutations**

Table S10: The dN/dS estimation for the branch leading to the *Thermus* virus NrdJm sequences yielded an over all dN/dS value of 17.5. For 40 positions, significant non-conservative mutations were found with a posterior probability >= 95%.
Supplemental file: Table_S10_Figure_1D_Non_Conservative.xlsx

To map the non-conservative in the NrdJm a homology model was generated vis Swissmodel Server applying the NrdJm from *Lactobacillus leichmannii* as template (PDB entry:1L1L). The model is attached as: TVNrdJm_1L1L.pdb

1. **Enzyme purification**

After harvesting the expression culture by centrifugation, cell pellets were stored at -20°C. Cell lysis was performed by a combined lysozyme/sonication procedure. The cell pellets were resuspended in Lysis buffer (Table S3) and incubated for 60 min at 8 °C and 50 RPM. Afterwards, the sample was sonicated three times 30 s with a Sartorius LABSONIC M^®^ (Amplitude 100 %, Cycle 0.6). Cell debris were removed by centrifugation at 15000 g for 60 minutes.

All chromatographic purification steps were performed on a Äkta FPLC 900^®^ system. The supernatant of the centrifugation was loaded on a 5 mL His-Trap FF^®^ column and washed with 20 mL His-Wash buffer (Table S3). The target enzyme was eluted with 20 mL His-Elute buffer (Table S3) and collected in a fraction collector. While the aforementioned steps were the same for both all enzymes, for the NrdJm was further purified by size exclusion chromatography while thioredoxin and thioredoxin reductase were subjected to desalting only.

The IMAC purified NrdJm was concentrated via Vivaspin^®^ 20 mL centrifugal concentrators and loaded on a Highload 16/60^TM^ Superdex 75^TM^ size exclusion column. The sample was eluted with SEC buffer (Table S3). To the eluted sample 20 % (v/v) glycerol was added. The samples were aliquoted, frozen in liquid nitrogen and stored at -80°C.

The IMAC purified thioredoxin and thioredoxin reductase were loaded on a Highprep 26/10^TM^ desalting column and eluted with SEC buffer (Table S3). To the eluted samples 20 % (v/v) glycerol was added. The samples were aliquoted, frozen in liquid nitrogen and stored at -80°C. Figure S1 shows the final sample of the purifications of all applied enzymes.


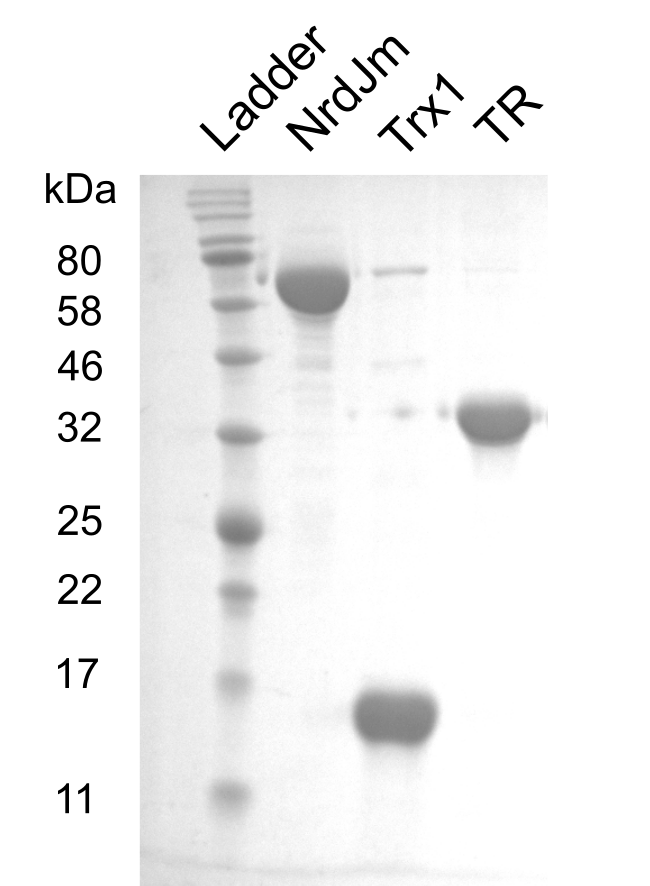


Figure S1: SDS-PAGE gel of the purified enzymes NrdJm, thioredoxin (Trx1) and thioredoxin reductase (TR).

1. **Analytical size exclusion chromatography**


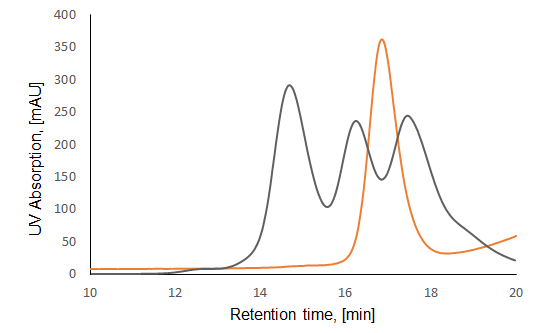


Figure S2: Analytical size exclusion chromatography of NrdJm (red) and Standards (black): with increasing retention time Aldolase 158 kDa, Conalbumin 75 kDa, Ovalbumin 43 kDa.

1. **Acticity assays - Raw data and UHPLC analytics**

| *Sample name* | *dGTP Peak area [ ]* | *dGTP concentration [mmol/L]* | *kcat [1/min]* | *Average kcat [1/min]* | *Standard deviation kcat [1/min]* |
| --- | --- | --- | --- | --- | --- |
| *B12 0 µM a* | 8417 | 0.003 | 0.04 | 0.03 | 0.01 |
| *B12 0 µM b* | 5700 | 0.002 | 0.03 |  |  |
| *B12 0 µM c* | 5386 | 0.002 | 0.03 |  |  |
| *B12 4 µM a* | 748065 | 0.227 | 3.78 | 3.95 | 0.13 |
| *B12 4 µM b* | 794987 | 0.241 | 4.01 |  |  |
| *B12 4 µM c* | 804903 | 0.244 | 4.06 |  |  |
| *B12 10 µM a* | 792346 | 0.240 | 4.00 | 3.40 | 0.44 |
| *B12 10 µM b* | 582134 | 0.176 | 2.94 |  |  |
| *B12 10 µM c* | 644691 | 0.195 | 3.26 |  |  |
| *B12 20 µM a* | 640729 | 0.194 | 3.24 | 3.21 | 0.13 |
| *B12 20 µM b* | 665031 | 0.201 | 3.36 |  |  |
| *B12 20 µM c* | 600867 | 0.182 | 3.03 |  |  |
| *B12 40 µM a* | 493188 | 0.149 | 2.49 | 2.83 | 0.31 |
| *B12 40 µM b* | 643049 | 0.195 | 3.25 |  |  |
| *B12 40 µM c* | 546571 | 0.166 | 2.76 |  |  |
| *B12 100 µM a* | 307170 | 0.093 | 1.55 | 1.68 | 0.20 |
| *B12 100 µM b* | 302235 | 0.092 | 1.53 |  |  |
| *B12 100 µM c* | 390031 | 0.118 | 1.97 |  |  |

Table S11: Raw data for Figure 2A: Effect of B_12_-concentration on ribonucleotide reductase activity. Enzyme concentration: 4 µM, Assay time: 15 min

| *Sample name* | *dGTP Peak area [ ]* | *dGTP concentration [mmol/L]* | *kcat [1/min]* | *Average kcat [1/min]* | *Standard deviation kcat [1/min]* |
| --- | --- | --- | --- | --- | --- |
| *25°C a* | 506747 | 0.154 | 5.12 | 4.89 | 0.21 |
| *25°C b* | 455894 | 0.138 | 4.60 |  |  |
| *25°C c* | 491275 | 0.149 | 4.96 |  |  |
| *30°C a* | 812052 | 0.246 | 8.20 | 8.54 | 0.30 |
| *30°C b* | 884522 | 0.268 | 8.93 |  |  |
| *30°C c* | 840926 | 0.255 | 8.49 |  |  |
| *40°C a* | 1875534 | 0.568 | 18.94 | 19.34 | 1.28 |
| *40°C b* | 2086132 | 0.632 | 21.07 |  |  |
| *40°C c* | 1783093 | 0.540 | 18.01 |  |  |
| *50°C a* | 2287252 | 0.693 | 23.10 | 23.87 | 1.20 |
| *50°C b* | 2272542 | 0.688 | 22.95 |  |  |
| *50°C c* | 2531303 | 0.767 | 25.56 |  |  |
| *60°C a* | 1383031 | 0.419 | 41.90 | 39.94 | 1.76 |
| *60°C b* | 1330212 | 0.403 | 40.30 |  |  |
| *60°C c* | 1241950 | 0.376 | 37.62 |  |  |
| *70°C a* | 1345765 | 0.408 | 40.77 | 39.86 | 0.72 |
| *70°C b* | 1287814 | 0.390 | 39.01 |  |  |
| *70°C c* | 1313426 | 0.398 | 39.79 |  |  |
| *80°C a* | 11416 | 0.003 | 0.12 | 0.08 | 0.03 |
| *80°C b* | 5957 | 0.002 | 0.06 |  |  |
| *80°C c* | 5766 | 0.002 | 0.06 |  |  |
| *90°C a* | 0 | 0.000 | 0.00 | 0.00 | 0.00 |
| *90°C b* | 0 | 0.000 | 0.00 |  |  |
| *90°C c* | 0 | 0.000 | 0.00 |  |  |

Table S12: Raw data for Figure 2B: Effect of temperature on ribonucleotide reductase activity. At all temperatures except for 60°C and 70°C: Enzyme concentration: 2 µM, Assay time: 15 min; At 60°C and 70°C: Enzyme concentration: 1 µM, Assay time: 10 min

| *Sample name* | *dGTP Peak area [ ]* | *dGTP concentration [mmol/L]* | *kcat [1/min]* | *Average kcat [1/min]* | *Standard deviation kcat [1/min]* |
| --- | --- | --- | --- | --- | --- |
| *pH = 5.0 MES a* | 70572 | 0.021 | 0.36 | 0.43 | 0.06 |
| *pH = 5.0 MES b* | 99548 | 0.030 | 0.50 |  |  |
| *pH = 5.0 MES c* | 85207 | 0.026 | 0.43 |  |  |
| *pH = 5.5 MES a* | 135369 | 0.041 | 0.68 | 0.72 | 0.10 |
| *pH = 5.5 MES b* | 169120 | 0.051 | 0.85 |  |  |
| *pH = 5.5 MES c* | 120713 | 0.037 | 0.61 |  |  |
| *pH = 6.0 MES a* | 280093 | 0.085 | 1.41 | 1.24 | 0.15 |
| *pH = 6.0 MES b* | 249629 | 0.076 | 1.26 |  |  |
| *pH = 6.0 MES c* | 206645 | 0.063 | 1.04 |  |  |
| *pH = 6.5 MES a* | 641412 | 0.194 | 3.24 | 3.19 | 0.09 |
| *pH = 6.5 MES b* | 647276 | 0.196 | 3.27 |  |  |
| *pH = 6.5 MES c* | 605700 | 0.183 | 3.06 |  |  |
| *pH = 7.0 MES a* | 937727 | 0.284 | 4.73 | 5.05 | 0.23 |
| *pH = 7.0 MES b* | 1037878 | 0.314 | 5.24 |  |  |
| *pH = 7.0 MES c* | 1026087 | 0.311 | 5.18 |  |  |
| *pH = 7.0 TRIS a* | 839587 | 0.254 | 4.24 | 4.52 | 0.22 |
| *pH = 7.0 TRIS b* | 897523 | 0.272 | 4.53 |  |  |
| *pH = 7.0 TRIS c* | 945960 | 0.287 | 4.78 |  |  |
| *pH = 7.5 TRIS a* | 1164461 | 0.353 | 5.88 | 5.46 | 0.31 |
| *pH = 7.5 TRIS b* | 1061977 | 0.322 | 5.36 |  |  |
| *pH = 7.5 TRIS c* | 1016328 | 0.308 | 5.13 |  |  |
| *pH = 8.0 TRIS a* | 1071600 | 0.325 | 5.41 | 5.17 | 0.20 |
| *pH = 8.0 TRIS b* | 1029880 | 0.312 | 5.20 |  |  |
| *pH = 8.0 TRIS c* | 973004 | 0.295 | 4.91 |  |  |
| *pH = 8.5 TRIS a* | 870640 | 0.264 | 4.40 | 4.11 | 0.21 |
| *pH = 8.5 TRIS b* | 796622 | 0.241 | 4.02 |  |  |
| *pH = 8.5 TRIS c* | 774062 | 0.234 | 3.91 |  |  |
| *pH = 9.0 TRIS a* | 570971 | 0.173 | 2.88 | 2.96 | 0.08 |
| *pH = 9.0 TRIS b* | 608275 | 0.184 | 3.07 |  |  |
| *pH = 9.0 TRIS c* | 581717 | 0.176 | 2.94 |  |  |

Table S13: Raw data for Figure 2C: Effect of the pH value on ribonucleotide reductase activity. Enzyme concentration: 4 µM, Assay time: 15 min

| *Sample name* | *dGTP Peak area [ ]* | *dGTP concentration [mmol/L]* | *kcat [1/min]* | *Average kcat [1/min]* | *Standard deviation kcat [1/min]* |
| --- | --- | --- | --- | --- | --- |
| *GTP dATP a* | 26935 | 0.008 | 0.14 | 0.12 | 0.01 |
| *GTP dATP b* | 20531 | 0.006 | 0.10 |  |  |
| *GTP dATP c* | 25798 | 0.008 | 0.13 |  |  |
| *GTP dTTP a* | 898777 | 0.272 | 4.54 | 4.55 | 0.23 |
| *GTP dTTP b* | 957562 | 0.290 | 4.83 |  |  |
| *GTP dTTP c* | 846362 | 0.256 | 4.27 |  |  |
| *GTP dCTP a* | 39139 | 0.012 | 0.20 | 0.07 | 0.09 |
| *GTP dCTP b* | 0 | 0.000 | 0.00 |  |  |
| *GTP dCTP c* | 0 | 0.000 | 0.00 |  |  |
| *UTP dATP a* | 17845 | 0.009 | 0.15 | 0.15 | 0.02 |
| *UTP dATP b* | 14600 | 0.007 | 0.12 |  |  |
| *UTP dATP c* | 21005 | 0.011 | 0.18 |  |  |
| *UTP dTTP a* | 0 | 0.000 | 0.00 | 0.00 | 0.00 |
| *UTP dTTP b* | 0 | 0.000 | 0.00 |  |  |
| *UTP dTTP c* | 0 | 0.000 | 0.00 |  |  |
| *UTP dCTP a* | no separation |  |  |  |  |
| *UTP dCTP b* | no separation |  |  |  |  |
| *UTP dCTP c* | no separation |  |  |  |  |
| *UTP dGTP a* | no separation |  |  |  |  |
| *UTP dGTP b* | no separation |  |  |  |  |
| *UTP dGTP c* | no separation |  |  |  |  |
| *CTP dATP a* | 125826 | 0.064 | 1.06 | 0.83 | 0.19 |
| *CTP dATP b* | 70084 | 0.035 | 0.59 |  |  |
| *CTP dATP c* | 100343 | 0.051 | 0.85 |  |  |
| *CTP dTTP a* | 0 | 0.000 | 0.00 | 0.00 | 0.00 |
| *CTP dTTP b* | 0 | 0.000 | 0.00 |  |  |
| *CTP dTTP c* | 0 | 0.000 | 0.00 |  |  |
| *CTP dGTP a* | 0 | 0.000 | 0.00 | 0.00 | 0.00 |
| *CTP dGTP b* | 0 | 0.000 | 0.00 |  |  |
| *CTP dGTP c* | 0 | 0.000 | 0.00 |  |  |
| *ATP dCTP a* | 85520 | 0.029 | 0.49 | 0.41 | 0.06 |
| *ATP dCTP b* | 67034 | 0.023 | 0.39 |  |  |
| *ATP dCTP c* | 62898 | 0.022 | 0.36 |  |  |
| *ATP dTTP a* | 0 | 0.000 | 0.00 | 0.00 | 0.00 |
| *ATP dTTP b* | 0 | 0.000 | 0.00 |  |  |
| *ATP dTTP c* | 0 | 0.000 | 0.00 |  |  |
| *ATP dGTP a* | 342853 | 0.118 | 1.97 | 2.20 | 0.29 |
| *ATP dGTP b* | 453659 | 0.156 | 2.61 |  |  |
| *ATP dGTP c* | 352251 | 0.121 | 2.02 |  |  |

Table S14: Raw data for table 2: Allosteric regulation analysis. Enzyme concentration: 4µM, Assay time: 15 min

| *Sample name* | *dGTP Peak area [ ]* | *dGTP concentration [mmol/L]* | *kcat [1/min]* | *Average kcat [1/min]* | *Standard deviation kcat [1/min]* |
| --- | --- | --- | --- | --- | --- |
| *DTT 0.5 mM a* | 820492 | 0.249 | 4.14 | 4.38 | 0.34 |
| *DTT 0.5 mM b* | 962977 | 0.292 | 4.86 |  |  |
| *DTT 0.5 mM c* | 819331 | 0.248 | 4.14 |  |  |
| *DTT 1 mM a* | 1146157 | 0.347 | 5.79 | 6.14 | 0.26 |
| *DTT 1 mM b* | 1234646 | 0.374 | 6.23 |  |  |
| *DTT 1 mM c* | 1266678 | 0.384 | 6.40 |  |  |
| *DTT 2 mM a* | 1469963 | 0.445 | 7.42 | 7.17 | 0.29 |
| *DTT 2 mM b* | 1339540 | 0.406 | 6.76 |  |  |
| *DTT 2 mM c* | 1450399 | 0.439 | 7.32 |  |  |
| *DTT 5 mM a* | 1414368 | 0.428 | 7.14 | 6.83 | 0.26 |
| *DTT 5 mM b* | 1354114 | 0.410 | 6.84 |  |  |
| *DTT 5 mM c* | 1289205 | 0.391 | 6.51 |  |  |
| *DTT 10 mM a* | 1048520 | 0.318 | 5.29 | 4.80 | 0.41 |
| *DTT 10 mM b* | 955991 | 0.290 | 4.83 |  |  |
| *DTT 10 mM c* | 847930 | 0.257 | 4.28 |  |  |
| *DTT 20 mM a* | 1101745 | 0.334 | 5.56 | 5.31 | 0.18 |
| *DTT 20 mM b* | 1016579 | 0.308 | 5.13 |  |  |
| *DTT 20 mM c* | 1035481 | 0.314 | 5.23 |  |  |
| *DTT 50 mM a* | 785327 | 0.238 | 3.97 | 3.71 | 0.30 |
| *DTT 50 mM b* | 653050 | 0.198 | 3.30 |  |  |
| *DTT 50 mM c* | 767330 | 0.232 | 3.87 |  |  |
| *DTT 100 mM a* | 761361 | 0.231 | 3.84 | 3.16 | 0.53 |
| *DTT 100 mM b* | 612070 | 0.185 | 3.09 |  |  |
| *DTT 100 mM c* | 504314 | 0.153 | 2.55 |  |  |
| *TCEP 0.5 mM a* | 349609 | 0.106 | 3.53 | 3.22 | 0.23 |
| *TCEP 0.5 mM b* | 313438 | 0.095 | 3.17 |  |  |
| *TCEP 0.5 mM c* | 295004 | 0.089 | 2.98 |  |  |
| *TCEP 1 mM a* | 638313 | 0.193 | 6.45 | 6.01 | 0.32 |
| *TCEP 1 mM b* | 565907 | 0.171 | 5.71 |  |  |
| *TCEP 1 mM c* | 580278 | 0.176 | 5.86 |  |  |
| *TCEP 2 mM a* | 847929 | 0.257 | 8.56 | 9.18 | 0.59 |
| *TCEP 2 mM b* | 987941 | 0.299 | 9.98 |  |  |
| *TCEP 2 mM c* | 891606 | 0.270 | 9.00 |  |  |
| *TCEP 5 mM a* | 783046 | 0.237 | 7.91 | 8.92 | 2.06 |
| *TCEP 5 mM b* | 1168103 | 0.354 | 11.80 |  |  |
| *TCEP 5 mM c* | 698914 | 0.212 | 7.06 |  |  |
| *TCEP 10 mM a* | 735666 | 0.223 | 7.43 | 7.54 | 0.89 |
| *TCEP 10 mM b* | 859477 | 0.260 | 8.68 |  |  |
| *TCEP 10 mM c* | 644234 | 0.195 | 6.51 |  |  |
| *TCEP 20 mM a* | 894733 | 0.271 | 9.03 | 8.84 | 0.58 |
| *TCEP 20 mM b* | 797298 | 0.242 | 8.05 |  |  |
| *TCEP 20 mM c* | 934519 | 0.283 | 9.44 |  |  |
| *TCEP 50 mM a* | 761854 | 0.231 | 7.69 | 7.72 | 1.03 |
| *TCEP 50 mM b* | 640965 | 0.194 | 6.47 |  |  |
| *TCEP 50 mM c* | 891393 | 0.270 | 9.00 |  |  |
| *TCEP 100 mM a* | 665893 | 0.202 | 6.72 | 6.07 | 1.44 |
| *TCEP 100 mM b* | 403294 | 0.122 | 4.07 |  |  |
| *TCEP 100 mM c* | 734072 | 0.222 | 7.41 |  |  |

Table S15: Raw data for Figure 3A: Effect of artificial reductant and reductant concentration on ribonucleotide reductase activity. For DTT experiments: Enzyme concentration: 4 µM, Assay time: 15 min; For TCEP experiments: 2 µM, Assay time: 15 min

| *Sample name* | *dGTP Peak area [ ]* | *dGTP concentration [mmol/L]* | *kcat [1/min]* | *Average kcat [1/min]* | *Standard deviation kcat [1/min]* |
| --- | --- | --- | --- | --- | --- |
| *Trx1 - yes TR - yes a* | 163434 | 0.050 | 4.95 | 5.30 | 0.34 |
| *Trx1 - yes TR - yes b* | 190163 | 0.058 | 5.76 |  |  |
| *Trx1 - yes TR - yes c* | 171688 | 0.052 | 5.20 |  |  |
| *Trx1 - no TR - yes a* | 13125 | 0.004 | 0.40 | 0.48 | 0.14 |
| *Trx1 - no TR - yes b* | 11998 | 0.004 | 0.36 |  |  |
| *Trx1 - no TR - yes c* | 22471 | 0.007 | 0.68 |  |  |
| *Trx1 - yes TR -no a* | 60777 | 0.018 | 1.84 | 1.34 | 0.36 |
| *Trx1 - yes TR - no b* | 33587 | 0.010 | 1.02 |  |  |
| *Trx1 - yes TR - no c* | 38083 | 0.012 | 1.15 |  |  |
| *Trx1 - no TR -no a* | 23651 | 0.007 | 0.72 | 0.56 | 0.21 |
| *Trx1 - no TR - no b* | 8737 | 0.003 | 0.26 |  |  |
| *Trx1 - no TR - no c* | 23045 | 0.007 | 0.70 |  |  |

Table S16: Raw data for Figure 3B: Allosteric regulation analysis. Enzyme concentration: 2 µM, Assay time: 5 min

Analytics for the ribonucleotide reduction reaction were performed via UHPLC as described in the main article. Table S2 shows the retention times of all substrates and reaction products.

| **Substrate** | **Retention time [min]** | **Product** | **Retention time [min]** |
| --- | --- | --- | --- |
| **ATP** | 5.5 | dATP | 6.2 |
| **CTP** | 8.9 | dCTP | 9.7 |
| **GTP** | 4.5 | dGTP | 5.0 |
| **UTP** | 4.7 | dUTP | 5.7 |

Table S17: Retention times of relevant analytes applying the HPLC methods described in the materials and methods section of the main manuscript.
